# Supplementary material for: Mesenchymal Stromal Cell Exosomes Mediate M2-like Macrophage Polarization through CD73/Ecto-5′-Nucleotidase Activity
Source: Pharmaceutics. 2023 May 13;15(5):1489. doi: 10.3390/pharmaceutics15051489 (PMC10220822; doi:10.3390/pharmaceutics15051489)
Supplement: Supplementary file 1 [file pharmaceutics-15-01489-s001.zip › pharmaceutics-2373675-supplementary.pdf]

Supplementary materials

Mesenchymal stromal cell exosomes mediate M2-like macrophage polarization through CD73/ecto-5′-nucleotidase activity

Kristeen Ye Wen Teo[1,2], Shipin Zhang[1,2], Jia Tong Loh[3], Ruenn Chai Lai[4], Hwee Weng Dennis Hey[1], Kong-Peng Lam[3], Sai Kiang Lim[4], Wei Seong Toh[1,2,5,6*]

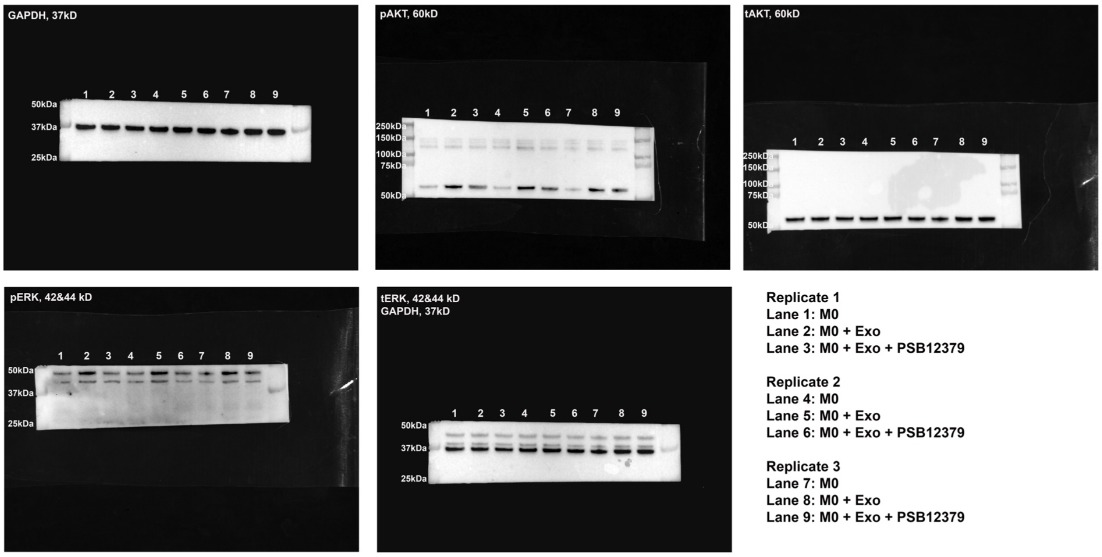

Figure S1: Individual blots for western blot analysis of AKT and ERK activation in primary macrophages treated with MSC exosomes in the presence or absence of CD73 inhibition by PSB12379.

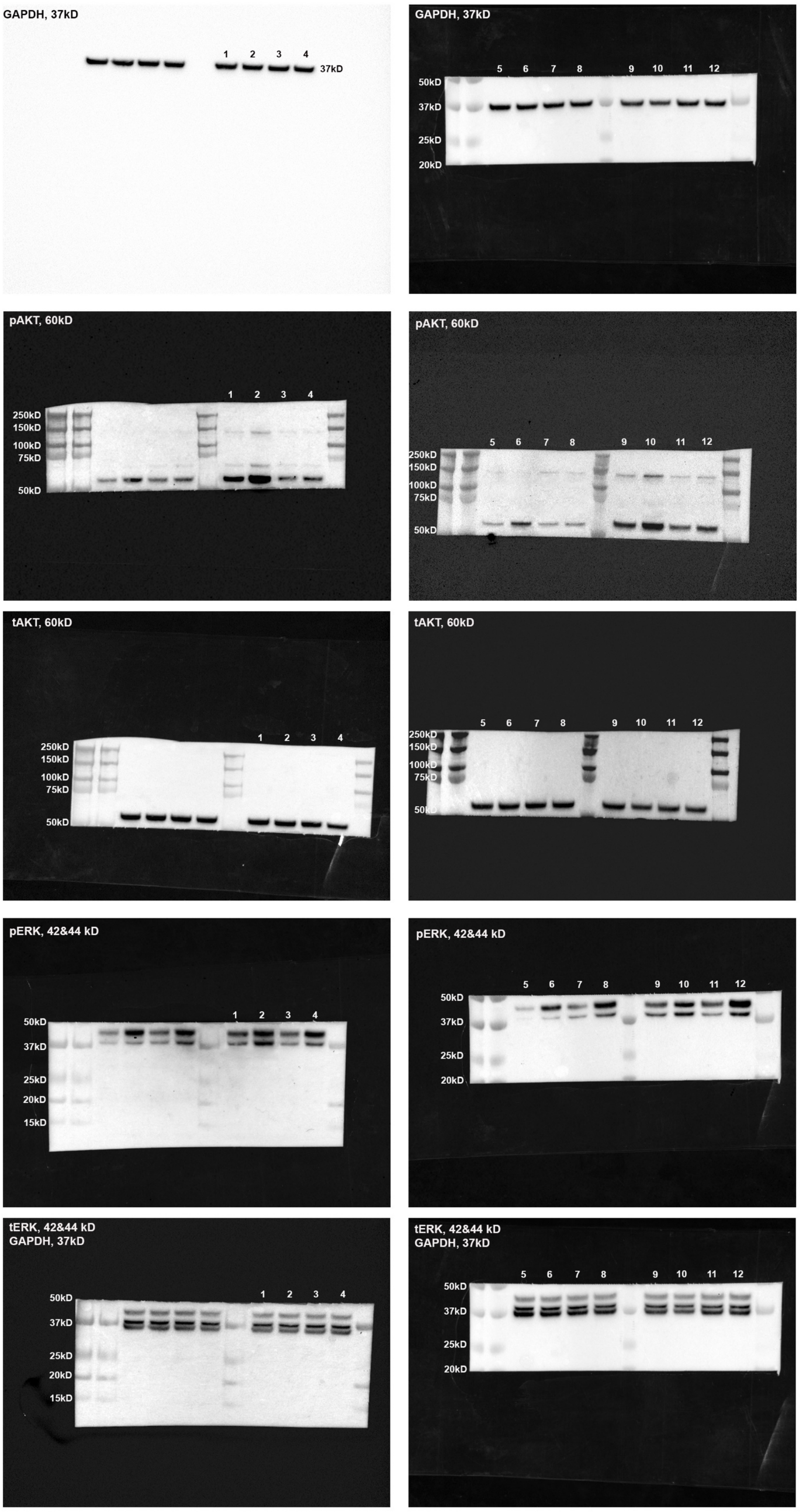

Replicate 1
Lane 1: M0
Lane 2: M0 + Exo
Lane 3: M0 + Exo + ZM241385
Lane 4: M0 + Exo + PSB1115

Replicate 2
Lane 5: M0
Lane 6: M0 + Exo
Lane 7: M0 + Exo + ZM241385
Lane 8: M0 + Exo + PSB1115

Replicate 3
Lane 9: M0
Lane 10: M0 + Exo
Lane 11: M0 + Exo + ZM241385
Lane 12: M0 + Exo + PSB1115

Figure S2: Individual blots for western blot analysis of AKT and ERK activation in primary macrophages pre-treated with A_{2A} receptor inhibitor (ZM241385) and A_{2B} receptor inhibitor (PSB1115) before exosome treatment.

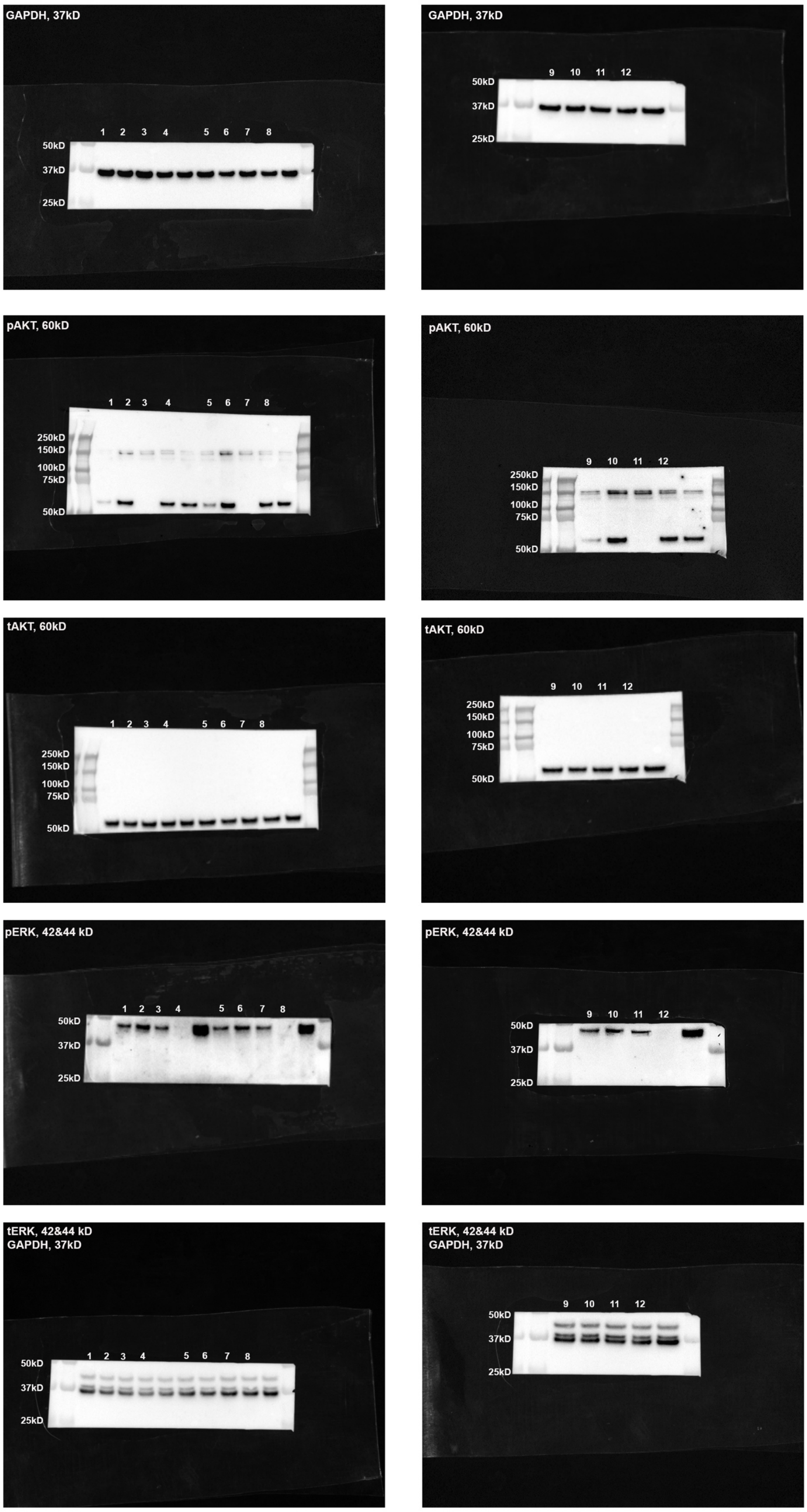

Replicate 1
Lane 1: M0
Lane 2: M0 + Exo
Lane 3: M0 + Exo+Wortmannin
Lane 4: M0 + Exo+U0126

Replicate 2
Lane 5: M0
Lane 6: M0 + Exo
Lane 7: M0 + Exo+Wortmannin
Lane 8: M0 + Exo+U0126

Replicate 3
Lane 9: M0
Lane 10: M0 + Exo
Lane 11: M0 + Exo+Wortmannin
Lane 12: M0 + Exo+U0126

Figure S3: Individual blots for western blot analysis of AKT and ERK activation in primary macrophages pre-treated with AKT inhibitor (Wortmannin) and ERK inhibitor (U0126) before exosome treatment.
